# Comments on the Death of Dr. Huidekoper

**Published:** 1902-01

**Authors:** 


### COMMENTS ON THE DEATH OF DR. HUIDEKOPER.

### RUSH SHIPPEN HUIDEKOPER.

THIS distinguished veterinarian died of a complication of diseases, hastened by pneumonia, at Philadelphia, on December 17th ult., and the profession of America has thus lost one of its most conspicuous representatives. Few men have entered upon their life-work better equipped for it than our deceased colleague. Possessed of a splendid constitution, a liberal education, great wealth and influence, a natural love for its truths and mysteries, he could easily command whatever his fancy dictated. A basic medical education placed him in an enviable position when he sought his veterinary degree from historic Alfort, and, not content with his stamp of approval, he entered the famous laboratories of Virchow, Koch, Chauveau, and Pasteur, to complete his technical training. Fortified with such advantages, it was not surprising that he should have made an impression in the struggling profession of America, and it may be said that he has held every post of honor that was within her gift. As editor, teacher, author, medical officer, associationist, and practitioner, he distinguished himself by his great learning and his devotion to her interests, and his death has cast a long shadow over the profession of the country.

Personally, Dr. Huidekoper was a companionable and genial gentleman, who made friends easily and held them firmly, by all of whom his death will be sincerely regretted.—*American Veterinary Review*, January, 1902.

We regretted very much to hear of the death of Dr. Huidekoper. We feel that we have lost a man of great worth to the veterinary profession. It seemed too bad that we should lose him just as this era of advancement for the profession was brightening.

S. B. NELSON, D.V.M., Pullman, Washington.

Yours and paper containing the sad news of the passing away of our esteemed friend and head of the profession received. We are aware of the great claims which Dr. Huidekoper enjoyed in dignifying a profession, of the popularity which accompanied him, and of the sincere affection with which the entire profession regarded him; of his untiring labors in endeavoring to bring recognition to a profession, of the sacrifices in the founding of an institution, and the ordeal through which he passed in his efforts, which must have operated as a serious blow in his life. He will stand out to future generations with distinctness.

S. E. WEBER, V.S., Shady Grove, Pa.

The loss to the general profession will be greatly felt, and particularly by the army branch of it, whose champions his noble wife and himself proved themselves.

R. B. CORCORAN, Vet., Presidio, San Francisco, Cal.

I am very sorry to learn of Dr. Huidekoper's death. I had the pleasure of meeting him at Detroit, in 1900, and conceived a very favorable opinion of him. His death is a loss to the entire profession.

F. TORRANCE, D.V.S., Winnipeg, Manitoba.

It is only a little while ago that I learned of the death of Dr. Huidekoper. Progressive veterinary medicine has lost its most ardent champion. I do regret it bitterly that I did not know of his illness, as it would have been a great satisfaction to me to cheer him with a heartfelt note while ill.

May his kindly spirit ever continue with all of us and the JOURNAL.

W. E. A. WYMAN, M.D., V.S., Milwaukee, Wis.

The death of your associate, Dr. Huidekoper, I mourn very deeply, having been a student under him in my freshman year at the New York College of Veterinary Surgeons. He examined us in anatomy while confined on his back in bed—an illustration of his faithfulness to duty and deep interest in his students.

J. HAYDEN STIMSON, V.S., Princeton, Mass.

I regret very much the death of Dr. Huidekoper, as I considered him a very valuable man for our profession.

M. JACOB, V.M.D., Knoxville, Tenn.

I was extremely sorry to learn of Dr. Huidekoper's death, and feel that the profession has sustained an incomputable loss thereby.

J. H. TIMBERMAN, V.S.

I was very sorry to hear of the death of Dr. Huidekoper. It is an irreparable loss to the veterinary profession.

ALEX. EGER, Chicago, Ill.

